# Genetic analysis of the cold-sensitive growth phenotype of *Burkholderia pseudomallei/thailandensis* bacteriophage AMP1

**DOI:** 10.1038/s41598-022-07763-7

**Published:** 2022-03-11

**Authors:** Andrey V. Letarov, Maria A. Letarova, Pavel A. Ivanov, Ilya S. Belalov, Martha R. J. Clokie, Edouard E. Galyov

**Affiliations:** 1 Winogradsky Institute of Microbiology RC Biotechnology RAS, Moscow, Russia; 2 Department of Genetics and Genome Biology, University of Leicester, Leicester, UK

**Keywords:** Bacteriophages, Prokaryote

## Abstract

Bacteriophages related to phage Bp_AMP1 are the most widely spread group of phages infecting *Burkholderia pseudomallei*—the causative agent of melioidosis. These viruses are also infective against the nonpathogenic host *Burkholderia thailandensis,* allowing experimental work with them without any special safety precautions. The indirect data as well as the results of the mathematical modelling suggest that the AMP1-like viruses may act as natural biocontrol agents influencing the population levels of *B. pseudomallei* in soil and water habitats in endemic regions. The cold sensitivity of the lytic growth (CSg) of these phages was suggested to be an important feature modulating the effect of viral infection on host populations in nature. We performed genetic analysis to determine the molecular background of the CSg phenotype of the AMP1 phage. The results indicate that CSg is not due to the lack of any function or product missing at low temperature (25 °C) but results in growth inhibition by a phage-encoded temperature-sensitive genetic switch. We identified phage ORF3 and ORF14 to be involved in the genetic determination of this mechanism.

## Introduction

Most environmental microbial communities are complex and diverse. Bacteriophages (phages) play important roles within such communities: they control bacterial population dynamics and trafficking genes and shape bacterial community structure. Bacteria and their phages have coevolved to engage in interactions that promote their mutual long-term survival. Bacterial physiology and bacterial population sizes may vary dramatically depending on environmental parameters, such as seasonal factors and associated physical conditions. It is therefore of much interest how different types of phages respond to such changes and thus manage to survive the often significant periods of unfavorable conditions that are associated, for example, with low abundance of their bacterial host. It is widely believed that bacteriophage adaptation takes place mainly via microevolutionary processes^1–3^. However, some phages clearly have physiological mechanisms that allow them to alter their life cycles in response to certain changes in the environment. For example, coliphages T4, T2 and lactophage p2 are among the viruses that have been shown to modify their adsorption rate in response to chemical cues (see^4^ for review). SPbeta–like temperate phages and phage VP882 have also been shown to switch between lysis and lysogeny in response to quorum-sensing (QS) signals from phage or host-encoded QS systems^5, 6^. The relative proportion of lysogenic decisions in most temperate phages, including the extremely well-studied phage λ, depends on nutrient availability (reviewed in Ref.^7^) and on the temperature^8^.

In previous work, when we assessed phages that infect the important tropical pathogen *Burkholderia pseudomallei* and the closely related nonpathogenic environmental bacterium *Burkholderia thailandensis* in the natural environment*,* we found that AMP1-like viruses were the most ubiquitous and widely spread phage that we could isolate on this host from in natural ecosystems^9^. This phage type was frequently isolated from extensive surveys over large areas of Thailand^10^.

A striking observation in the characterization of AMP1 and related phages is that they are highly responsive to the temperature at which they infect their host. They behave like lytic predatory phages at temperatures of 37 °C but in contrast do not lyse their hosts at lower temperatures of 25 °C. When incubated at 37 °C, AMP1 phage forms clear plaques on both *B. thaliandensis* and *B. pseudomallei* lawns^9^ and causes seemingly complete lysis of the liquid cultures of these bacteria. However, at 25 °C, the growth of the AMP1 phage is inhibited^9^. It was suggested that at low temperature, the phage enters the lysogenic or pseudolysogenic state. We reported the generation of a lysogen that was stable at 25 °C but lysed with liberation of the phage at 37 °C^9^. Although the integration of the AMP1 phage could not be detected^9^, a putative integrase-encoding gene adjacent to the incomplete tRNA gene (sequence of this fragment is identical to that of tRNA-Arg and tRNA-Ser from *Burkholderia*) is present in the AMP1 phage genome, and such sequences are generally considered to be a genetic marker of the temperate lifestyle. The cold-sensitive growth (CSg) phenotype of AMP1-related phages is a potentially important feature that may influence the ecology of *B. pseudomallei* and AMP1-like phages in natural habitats in regions of melioidosis endemism, such as northeastern Thailand. These ideas, initially based on our observations from in vitro studies of the bacteria-phage interactions, are supported by mathematical modeling, which suggested that temperature-sensitive switching between supposed lysogeny and lytic development of the AMP1-like phages may cause seasonal variations in bacterial abundance and thus melioidosis cases observed in some areas of NE Thailand^11,12^. If this is indeed the case, understanding the mechanisms of interactions of *Burkholderia* host with AMP1 phage is clearly important for further analysis of the possible roles of phage infection in *B. pseudomallei* dynamics in nature.

Although the CSg phenotype of AMP1 phage was rigorously documented, it was unclear which component of the phage-host system, the bacterium or the virus, responds to temperature. It is also unclear whether phage growth is somehow downregulated at lower temperatures or if the CSg phenotype is due to a lack of an essential function (either viral or bacterial) under these conditions.

To address these mechanistic aspects and advance the understanding of the cause of temperature responsiveness in AMP1 phages, we selected and characterized a set of phage mutants that were insensitive to temperature. Our assessment of these mutants allowed us to establish that the CSg phenotype is indeed mediated by phage-encoded factors. Furthermore, we demonstrate that phage integrase, and thus classical integration-based temperature-responsive lysogeny, is not essential for the CSg phenotype of AMP1.

## Materials and methods

### Bioinformatic analysis

To find all available viruses and putative prophages related to AMP1, we used a tblastn search. For this search, we used the AMP1 integrase as the first query and the concatenated sequence of all other proteins encoded by the AMP1 genome as the second query. Two blast outputs were intersected, resulting in 6 viral genomes and 10 bacterial genomes containing putative integrated prophages (three phage genomes AMP2–4 closely related to AMP1 were excluded from our analysis). Each sequence was searched for tRNA with tRNAscan-SE^13^. The genomic content of AMP1 and 16 related (pro)phages was visualized with Clinker v0.0.21^14^.

All pairwise comparisons of the amino acid sequences were conducted using the Genome-BLAST Distance Phylogeny (GBDP) method^15^ under settings recommended for prokaryotic viruses^16^. The resulting intergenomic distances were used to infer a balanced minimum evolution tree with branch support via FASTME, including SPR postprocessing^17^. The distance between sequences was computed as the ratio of identical amino acid residues to the total length of blastp high-scoring segment pairs. Branch support was inferred from 100 pseudobootstrap replicates. The tree was rooted at the midpoint^18^ and visualized with FigTree^19^. Taxon boundaries at the species, genus and family levels were estimated with the OPTSIL program^20^, the recommended clustering thresholds^16^ and an F value (fraction of links required for cluster fusion) of 0.5^21^.

### Bacterial and phage strains and their propagation

Bacteriophage AMP-1 and *B. thailandensis* strains E264 and DW503^22^ were obtained from the collection of our laboratory (UoL, Leicester, UK). Bacteria were propagated on conventional LB medium: (10 g tryptone (Amresco, Belgium), 5 g yeast extract (Amresco, Belgium) 10 g NaCl, deionized water up to 1 L). This medium was supplemented with 15 g of Bacto-agar for the plates or with 6 g of Bacto-agar (Difco, USA) for soft medium used for the double-layer phage titration.

- Bacteriophage titration was performed with the standard double-layer protocol^23^. The host strain DW503 was used for phage propagation. Strain E264 was used for all the biological experiments. The efficiency of the phage plating (EOP) at low temperature was calculated as the biological titer of the phage stock obtained with the plates incubation at given temperature divided by the titer obtained with the plates incubation at 37 °C—the conditions yielding the maximal titer values for AMP1 phage and its mutants.

To propagate the phage, a single plaque was transferred from a fresh plate into 3–50 mL of the mid-log liquid culture of strain DW 506.1 (OD_600_ = 0.2), and the culture was incubated at 37 °C with vigorous agitation until lysis was visible or overnight. Then, 0.02% chlorophorm was added, and the lysate was vortexed. The cell debris was removed by centrifugation at 12,000×*g* for 10 min. Typically, such a procedure yielded a lysate with a biological titer of approximately 10^10^ PFU mL^−1^.

*The viricidal tea extract* was prepared as described in Ref.^24^. Briefly, 10 g of black leaf tea («The crown of the Russian Empire» tea, Russia, was purchased in a local food store) was infused with 100 mL of boiling deionized water and then incubated at 55 °C for 30 min. The extract was filtered through sterile filter paper. The tea extract was kept at + 4 °C and remained active for 1 week. The precipitate that forms after the cooling down of the extract was left unremoved.

### Generation of the independent CT mutants

AMP-W phage was plated at 37 °C to obtain isolated plaques. Several plaques were used to prepare the phage stocks, and the wild-type CSg phenotype was confirmed for each of these stocks. Since each of the plaques was grown from (presumably) a single virion of the CSg genotype, the CT mutants emerging in each stock should be of independent origin. To select such mutants, the stocks were plated at 25 °C on *B. thailandensis* lawn for selection. One CT plaque per initial stock was taken, and the phages were purified by repeated plaque isolation at 25 °C and amplified in liquid cultures at 37 °C.

### Plaques PCR screening for Spl1-like deletion

To check the phage plaques for presence of the Spl1-like deletion the fragments of the soft agar with the plaques were cut out of the plate with a sterile bacteriological loop and transferred into 0.3 mL of deionized water in the Eppendorf tubes. The samples were vortexed, incubated for 2 h at room temperature for phage extraction and then centrifuged at 12,000×*g* in a table-top microcentrifuge for 5 min. One microliter of the supernatant was used as a template in 20 μL PCR reaction with the primers, flanking the deletion in the Spl1 mutant genome: Spl-D (5′-CACAAGGAGGTCAACATGCA) and Spl-R (5′-CCAGCTTTGCGATGATCTC) using Taq-polymerase (SibEnzyme, Russia). The PCR program was: 95 °C—1 min followed by 30 cycles of 95 °C—30 s, 58 °C—30 s, 72 °C—1 min and by 1 min at 72 °C for post-extension. This reaction yielded ~ 300 b.p. product if the deletion was present and ~ 1600 b.p. in case the genome with the wild-type length of the region was used as a template.

### Phage DNA isolation and sequencing

To isolate phage DNA, 35 mL of the lysate was treated with DNAse (Biolot T, St. Petersburg, Russia; 0.005 mg mL^−1^ for 30 min at room temperature) and centrifuged in a bucket rotor at 70,000×*g* for 1 h to pellet down the phage particles. The pellet was resuspended in 0.5 mL of physiological saline, and the DNA was isolated by the conventional phenol-extraction protocol followed by ethanol precipitation^25^. The jellyfish-like DNA precipitate was trapped by a sterile bacteriological loop, rinsed in 70% ethanol and in water and dissolved in 100 μL of TE buffer.

The DNA was submitted for commercial genomic sequencing to MicrobesNG company (Birmingham, UK) using Illumina technology. The sequencing company assembled all the genomes, and all the phages yielded a single contig.

## Results and discussion

### AMP1-encoded integrase is not required for CSg phenotype

In our previous work, it was suggested^9^ that the CSg phenotype reflects the temperature sensitivity of the lysogenic decision, coupled with temperature-sensitive prophage induction. Notably, a similar mechanism has been described in some other phages. For example, phage λ lysogenizes almost all the infected cells at 25 °C, but the frequency of lysogenization drops to below 10% at 37 °C. This phenomenon is explained by the increased stability of the CII protein, which is the key transcription regulator of the lysogenic pathway^8^. In part, this increased stability is due to the enhanced translation of the cIII gene encoding the CII-stabilizing protein CIII. Temperature-dependent translation of the CIII messenger is ensured by RNA-thermometer control of cIII gene ribosome binding site accessibility^26, 27^.

**Figure 1:**
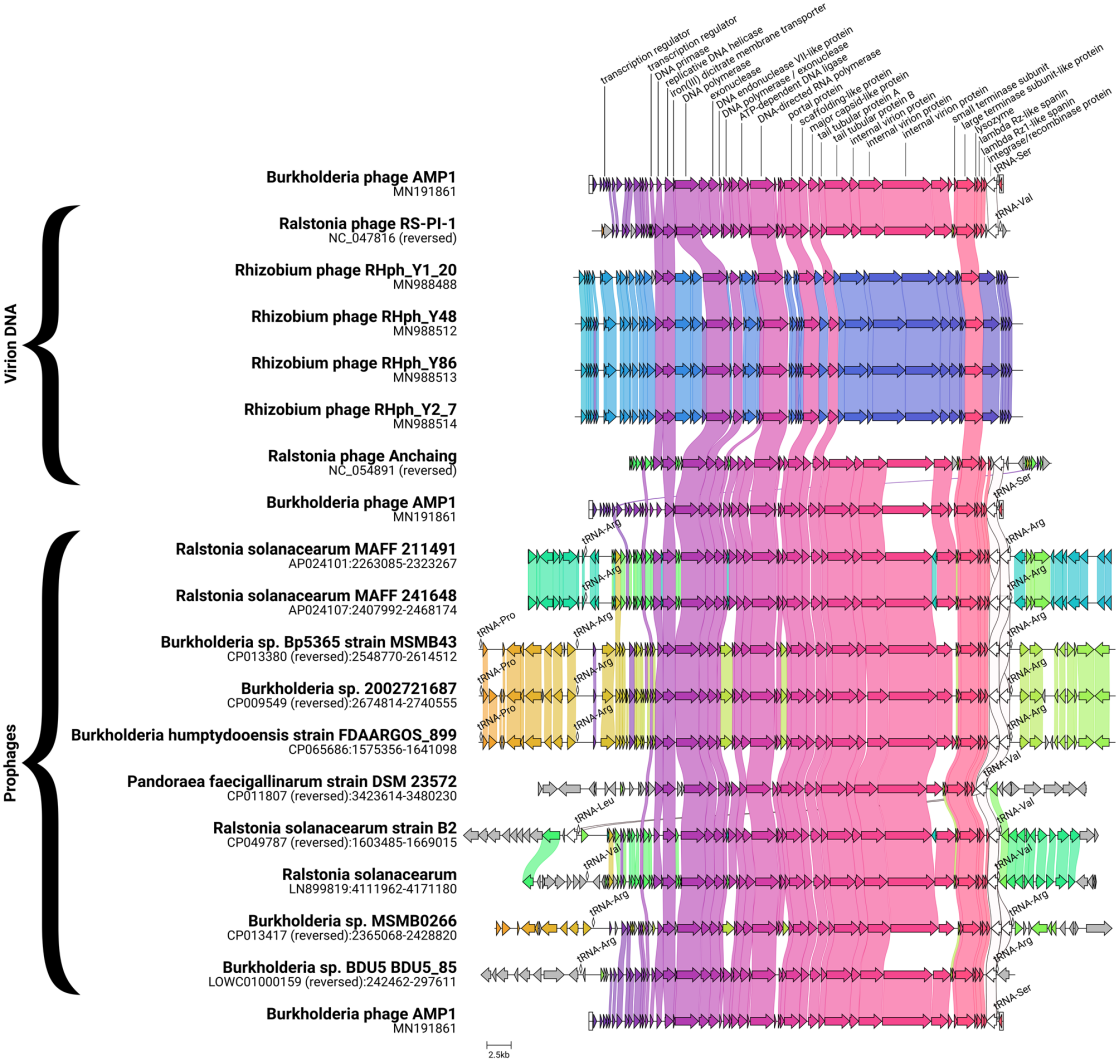
AMP1 related phages and putative prophages. Genes encoding proteins having more than 30% identical amino acid residues are linked. The color code for links and genes highlights sequence homology. Small diamonds indicate tRNA genes. Terminal repeats annotated in the phage genomes are boxed.

The bacteriophage AMP1 is distantly related to the T7 coliphage, a well-studied virulent virus. However, the AMP1 genome contains a putative integrase-encoding gene adjacent to the incomplete tRNA gene, and the presence of such sequences is generally believed to be a marker of the temperate virus. tRNA genes are frequently used as *att* sites by temperate phages^28^. These features indicate that the AMP1 phage is indeed capable of accessing the temperate life cycle, at least under some conditions. We performed a tblastn search (see “Materials and methods” section for details) to find potential AMP1-like sequences in the GenBank database. This search resulted in 6 viral sequences (three bacteriophages, AMP2–4, very closely related to AMP1, were excluded from the analysis) and 10 sequences of the potential prophages integrated into bacterial genomes. Interestingly, all these prophages were found to be flanked by repeated tRNA genes (Fig. 1), which is compatible with the hypothesis that tRNA genes are used as *att* sites by AMP1-related bacteriophages to integrate into bacterial genomes.

**Figure 2:**
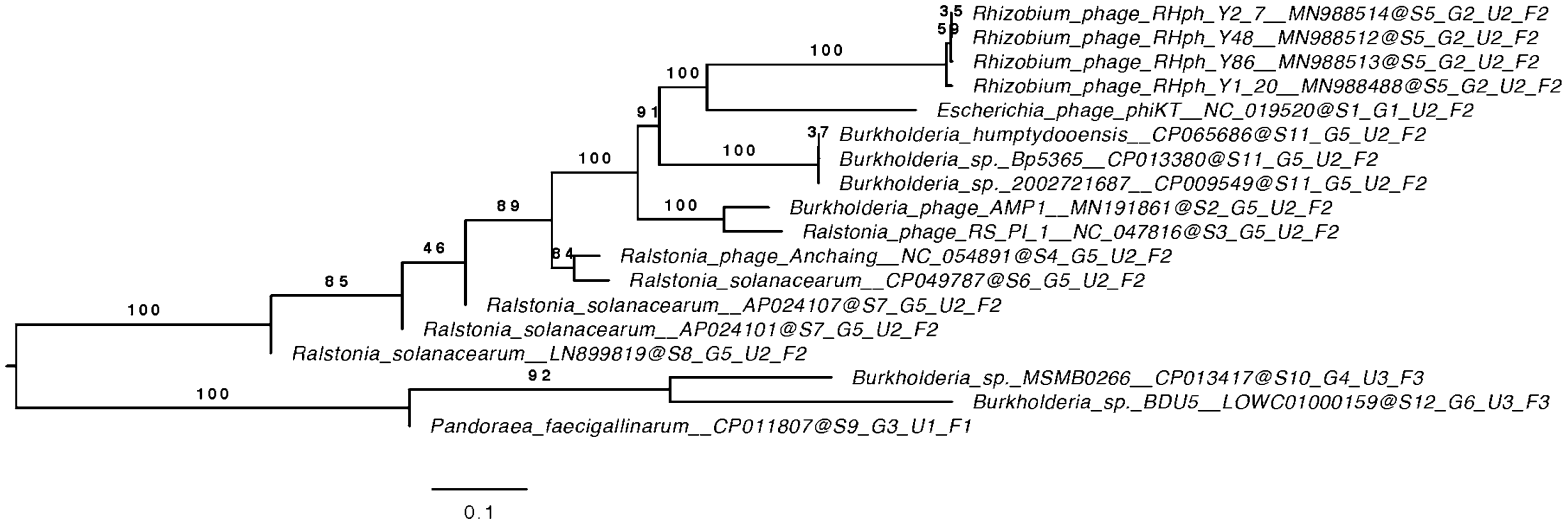
Phylogenomic GBDP tree of AMP1-related prophage sequences. The numbers above branches are GBDP pseudobootstrap support values from 100 replications. The branch lengths of the resulting VICTOR trees are scaled in terms of the respective distance formula used. OPTSIL clustering yielded fifteen species clusters. Eight, four and four clusters at the genus, subfamily, and family levels, respectively.

Putative AMP1-related prophages were found in *Burkholderia* genomes as well as in the genomes of *Ralstonia, Pandoraea* and *Rhizobium* (Fig. 2).

However, it is currently not clear how the genome organization of AMP1-like phages that are distantly related to the virulent coliphage T7^9^ is compatible with prophage maintenance. The induction of AMP1-related viruses from *B. pseudomallei* environmental isolates was also reported^10^. However, it is not clear if these isolates were indeed lysogenic, harboring a repressed prophage, or they were pseudolysogenic associations (also sometimes referred to as carrier state cultures) described for several phage-host systems^29–32^. Although the observed integration of the AMP1 genome into the host chromosome may occur by chance due to general recombination between a defective AMP1-like genome and chromosomally encoded tRNA genes, it cannot be excluded that it occurs as a part of the bacteriophage life cycle.

**Figure 3:**
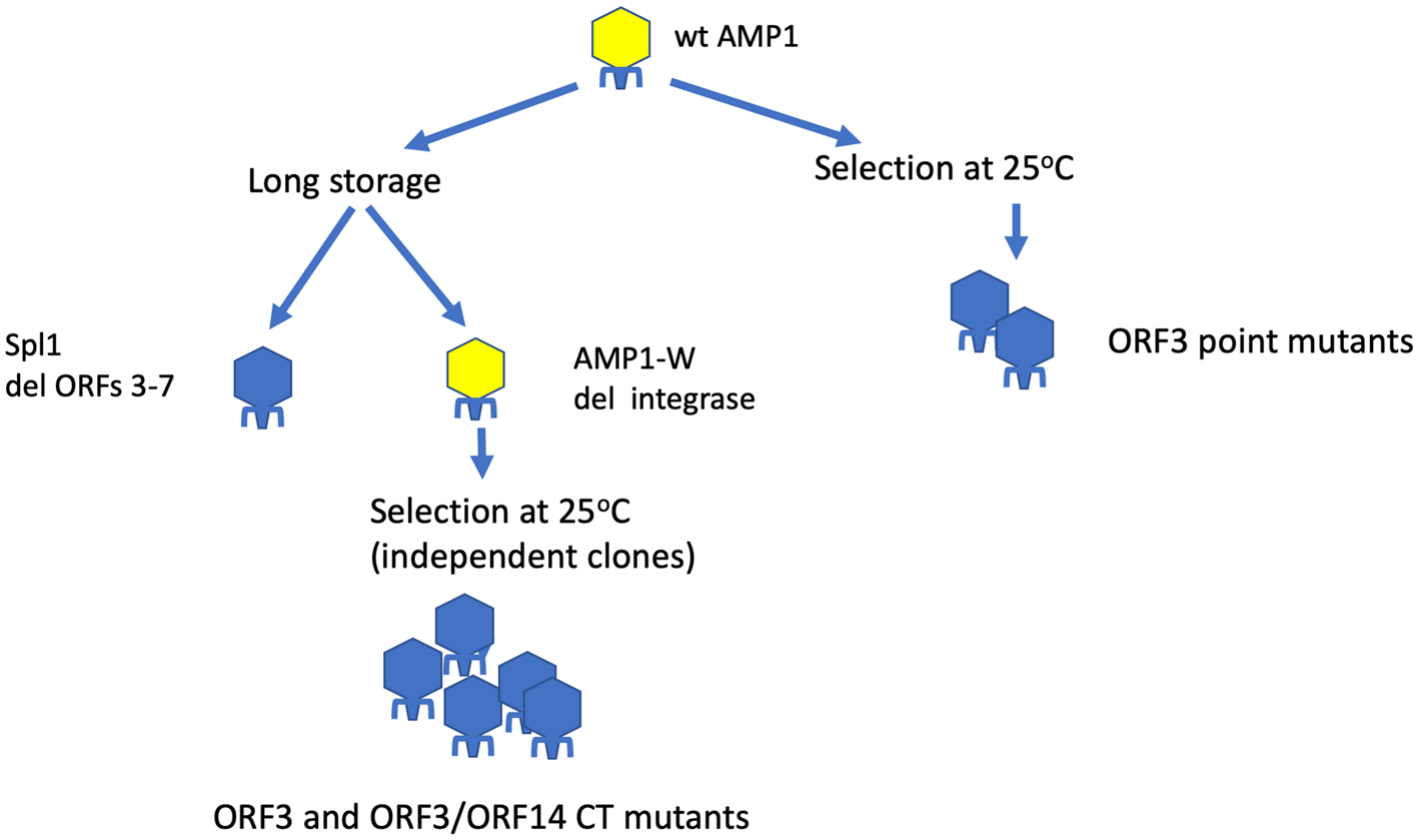
The genetic analysis workflow. Bacteriophage lineages featuring the CSg phenotype are shown in yellow, the cold-tolerant mutants are shown in blue.

Therefore, the potential involvement of phage-encoded integrase in the CSg phenotype is of particular interest. To reveal the possible involvement of the integrase and other phage genes into the determination of the CSg phenotype we selected and sequenced a set of the bacteriophage AMP1 mutants (the workflow of this genetic analysis is shown on the Fig. 3).

**Figure 4:**
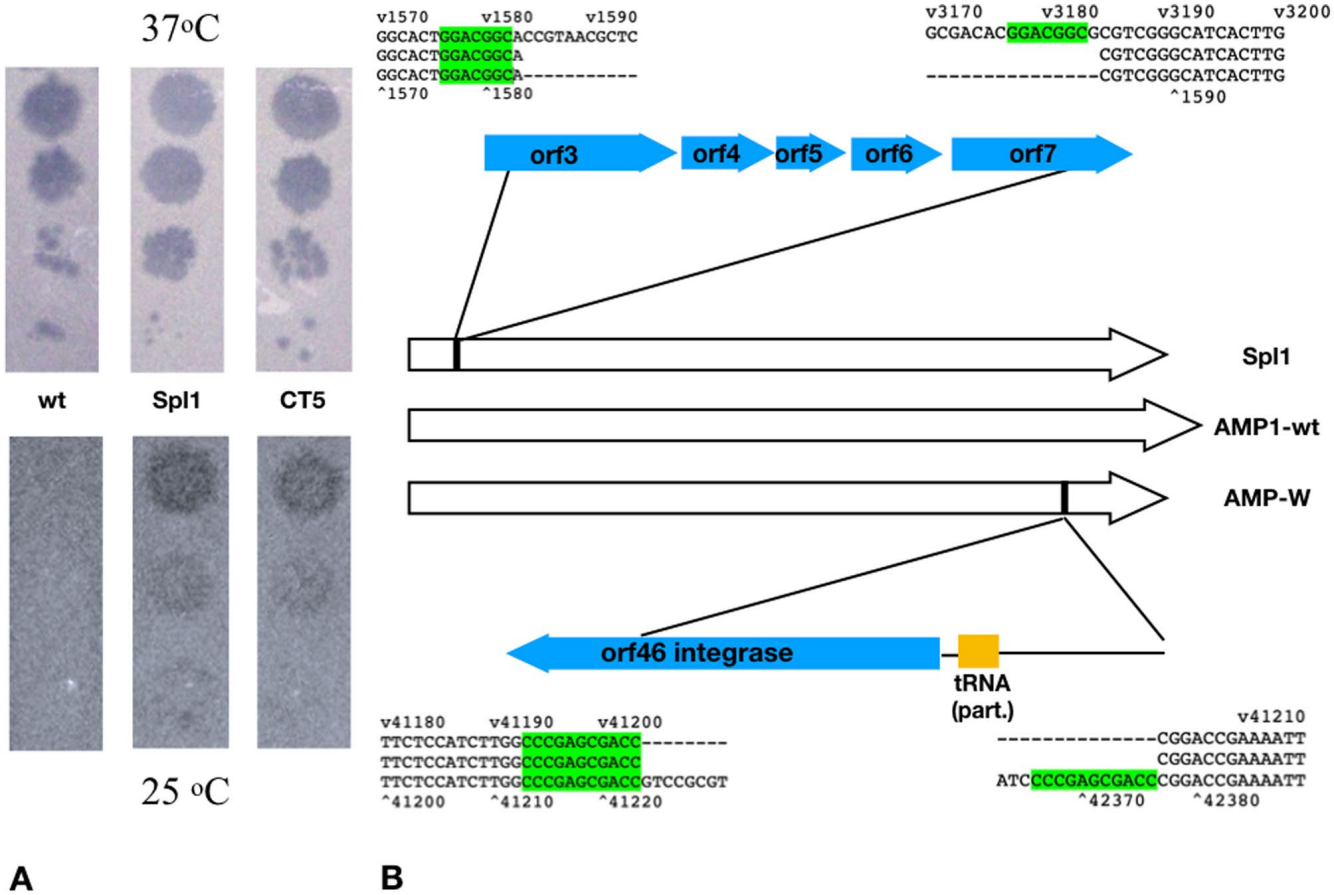
(**A**) Growth of the bacteriophage AMP1 wild-type (wt) strain and cold-tolerant mutant strains Spl1 and CT5 at 37 °C and 25 °C. (**B**) Schematic representation of the deletions that occurred in the bacteriophage strains AMP-W and Spl1. The short repeats flanking the deletions are highlighted in green.

It has been reported that for some phages, genetic instability may occur during long-term storage^33^. In an attempt to isolate spontaneous AMP1 phage mutants with altered phenotypes, we examined the phages from within a 3-year-old AMP1 phage lysate that had been stored at 4 °C. We titered this phage stock on the *B. thailandensis* DW506.1 lawn with incubation at 37 °C. Very few plaques were obtained, and the clearest five plaques were examined further. Each plaque was used to prepare a phage lysate, and their phenotypes were assessed. Interestingly, only two of the five phages showed the wild-type-like CSg phenotype, with high phage titers when incubated at 37 °C and no plaques at room temperature (23–25 °C). The remaining three phages formed plaques at both 37 °C and 25 °C, with an efficiency of plating of 0.1–0.3 at lower temperature compared to that at 37 °C (Fig. 4A), suggesting that these phages have lost the temperature-responsive phenotype.

To assess genetic determinants of these temperature-insensitive phages and that of the 3-year-old phages with the original temperature-sensitive phenotype, phage genomic DNA from each of the phages was isolated and sequenced. The assembled sequences were aligned with the reference genome sequence of AMP1 (wild type, *wt*). Surprisingly, both sequences contained sizeable deletions compared to the original deposited AMP1 sequence, but in addition to the deletions, no other alterations were seen. The mutant phage with the “normal” CSg phenotype is henceforth denoted AMP-W. It carried a deletion between nucleotide positions 41,224 and 42,376 (coordinates by the revised AMP1 genomic sequence, GenBank MN191861.1^34^). This deletion included most of the genes encoding integrase and the incomplete tRNA gene (Fig. 4B). These results suggest that integrase function is not essential in establishing CSg phenotype of AMP1.

### ORF3 is involved in CSg phenotype

The phage that demonstrated the cold-tolerant growth (CTg) phenotype was named ‘Spl1’. The genomic sequence of Spl1 phage contained an intact integrase locus; however, it had a deletion (Fig. 4B) between nucleotide positions 1584 and 3184 (AMP1 wild-type coordinates). This deletion affects five ORFs, three of which are fully deleted (Fig. 4B).

Interestingly, in both AMP-W and Spl1, the deleted regions were flanked in the wild-type sequence by short directly repeated motifs (CCCCGAGCGACC and GGACGGC, respectively; see Fig. 3B). Most likely, this is suggestive of the existence of strong recombination activity in the *B. thailandensis*-AMP1 system that can mediate crossover through such short regions of homology. Notably, both mutants were identified without selection for any particular phenotype with respect to growth at different temperatures, although stability upon storage may also act as a selective factor allowing enrichment of mutants carrying deletions.

The efficiency of plating of the Spl1 mutant was slightly reduced at lower temperatures compared to that at 37 °C, which may be related to some bacterial or phage physiological limitations that are not crucial for virus multiplication.

Bringing these two observations together, the phenotypes of the mutants AMP-W and Sp11 indicate that the temperature-responsive phenotype observed in the wild-type AMP1 phage is not controlled by the bacterial host or by biophysical constraints, such as temperature-sensitive folding of an essential phage protein, but is determined by a virus-encoded mechanism.

To further investigate factors involved in the temperature responsiveness of AMP1, we attempted to select other temperature-insensitive phage mutants capable of growing as plaques at 25 °C. We plated a fresh culture of the AMP1 wild-type phage at a high titer on *B. thailandensis* lawn at 25 °C. The efficiency of plating was approximately 10^–6^, which is consistent with data published previously^12^. Ten resulting plaques were purified by repeated plaque isolation at 25 °C, the lysates were grown, and their CT phenotypes were confirmed. PCR screening of these lysates for Spl1-like deletion revealed that none of them harbored such a polymorphism, indicating that a novel set of phage mutants with the CTg phenotype was obtained.

Genomic DNAs from two of the mutant phages were sequenced. Our analysis of the sequences revealed 3 SNPs in each of the genomes that cause a changed codon and thus amino acid substitutions in ORF 3 (T11A, Q56P, F57 L). This is of particular interest because it is consistent with the gene being replaced in the Spl1 mutant, thus further indicating the importance of this gene or the encoded protein for the CSg phenotype.

**Figure 5:**
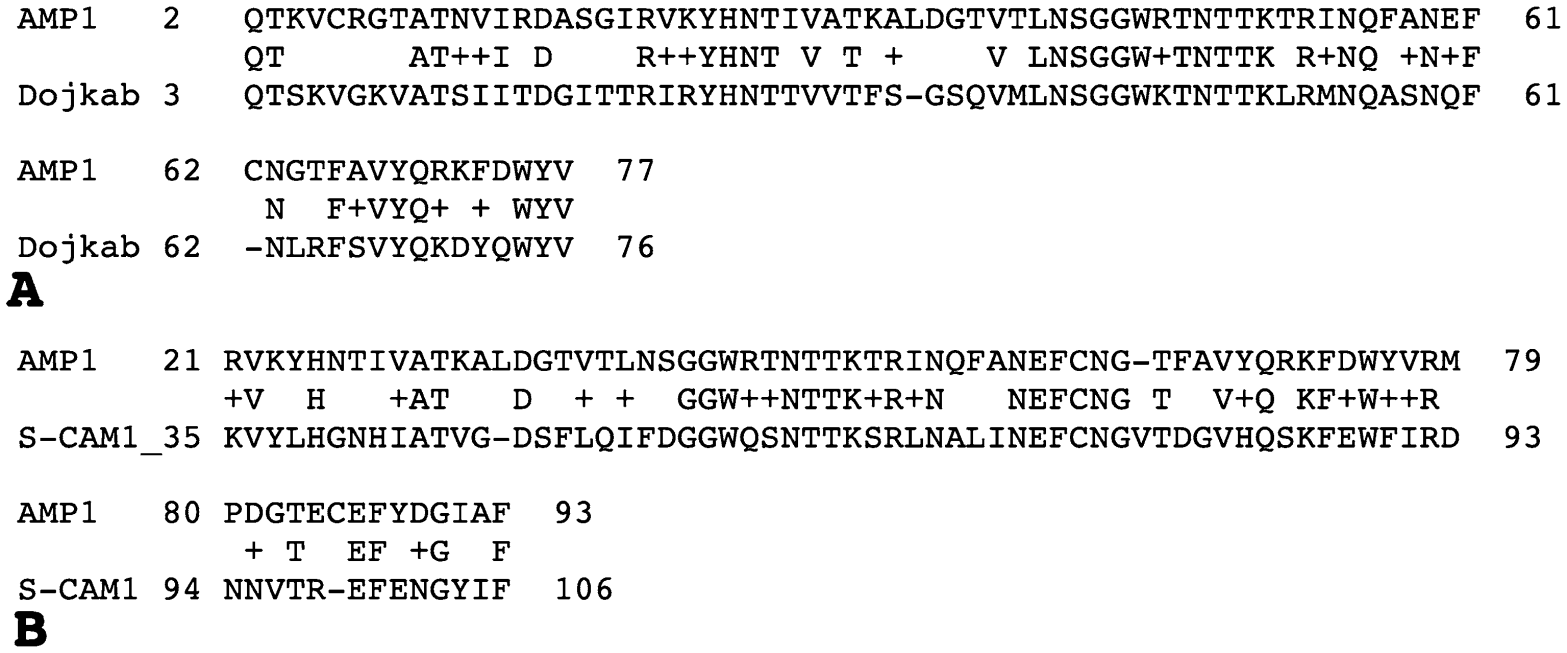
(**A**) BlastP alignment of the phage AMP1 ORF14 product and Candidatus Dojkabacteria bacterium protein (protein sequence ID: MBE0573628.1). (**B**) BlastP. alignment of the phage AMP1 ORF3 protein and ORF70 from the cyanobacterial myovirus S-CAM1.

A bioinformatic analysis did not identify any putative functional roles for the 107 aa product of ORF3, although a BlastP search revealed that it shares significant sequence similarity (a.a. identity level above 45%) with hypothetical proteins found in several bacterial genomes (see Fig. 5A for best hit) and, interestingly, in some cyanobacterial myoviruses (exemplified at Fig. 5B).

Our results suggest that this protein is involved in the regulation of AMP1 genome expression at different temperatures.

### Characterization of additional temperature-sensitive mutants derived from a phage lacking integrase

In the final set of experiments, we attempted to identify other potential components of CSg control by examining a set of phage mutants derived from the integrase-free mutant AMP-W. This allowed us to avoid any possible interference from the lysogeny phenotype. We obtained 7 independent CT mutants of the AMP-W phage originating from the phage stocks grown starting from isolated phage plaques. Genomic DNA from six mutants was isolated and sequenced.

The comparison of sequences of the mutant phages with the previously published AMP1 genome revealed several polymorphisms (Table S1). Since the mutant phage clones were of completely independent origin, we considered that the probability of identical mutations being responsible for the observed phenotype was low. Therefore, we excluded the polymorphisms present in all the mutants from consideration since they were presumably present in the phage culture used to obtain initial phage plaques before selection at lower temperature was applied. Since the abovementioned Spl and ORF3 mutants all have morphogenetic proteins identical to the wild type and are capable of successfully infecting host cells at 25 °C, we concluded that the CSg phenotype is due to the inhibition of intracellular phage development and not to poor phage attachment/infection onset at lower temperatures. This conclusion is in agreement with the abovementioned data on low-temperature infection by the CSg phage and with the fact that the mutations in ORF3, located in the early gene cluster, may be sufficient for the CTg phenotype. Based on these reasons, we decided to exclude polymorphisms seen in the morphogenetic genes from the analysis. The remaining mutations are listed in the Table 1.

**Table 1:** Polymorphisms in the genomic sequences of the CT mutants putatively involved in cold-tolerant phenotype determination.

Mutants ID	Polymorphisms (coordinates by AMP1 wild type)									
	A1467C	1615–16 ins C	A1667C	del1688-1722 (35 bp)	del1728-1733 (6 bp)	A1740G	ins4963-64 (135 bp)	Indels between 6531–78	C6686T	16,077–78 insertions
CT2	–	ORF3 frameshift after 47 aa	–					–	–	ORFf25 ins 129 bp ins 43 a.a
CT5	–	–	–			ORF3 D89G		ORF14, del 69–84	–	ORF25 ligase duplication 15,898–16,077 ins 45 aa
CT6	SD of ORF3 AGGcGG	–	–	–	–	–	orf10 ins 45 aa 67–68	ORF14 –//–	–	–
CT9	–	–	–	ORF3 del71–83, frameshift	–	ORF3 D89G		ORF14 aa 73–91 replaced by 48 aa	–	–
CT12	–	–	ORF3 T64P	–		–	–	–	interge-nic spacer	–
CT13	–	–	–	–	ORF3 del84–85 (EC)	–	–	–	–	ORF25 ins 135 bp (two adjacent duplications)

The loci affected by these polymorphisms and types of alterations are given in the cells. Designations: *SD* Shine-Dalgarno ribosome-binding site, *del* deletion, *ins* insertion, *indels between* multiple nucleotide deletions and insertions in the indicated sequence region, “–” the polymorphism is not present in the corresponding mutant.

### ORF14 is the second candidate gene involved in the CSg phenotype

Consistent with data showing that ORF 3 determines the temperature phenotype, in all but CT6 mutant phages, either nucleotide substitutions resulting in at least one amino acid substitution or in-frame deletions were observed in ORF3 (Table 1). In CT6, the Shine-Dalgarno sequence of ORF3 was altered by the point mutation turning AGGcGG (compared to AGGAGG in the wild type). This may decrease the translation of this ORF, although it is not clear to what extent this effect is significant for the phenotype.

Of interest, CT6, in which the ORF3 coding sequence is intact, as well as two other mutant phages, CT5 and CT9, carried sequence alterations in ORF14, suggesting that this protein may also be involved in the CSg control of the wild-type phage. The blastP search with the ORF14 aa sequence as a query yields multiple hits to hypothetical proteins from different bacteria with aa identity levels up to 70%, including bacterial species in which our analysis did not identify any AMP1-related prophage (see Fig. 5B for an example). The HHpred search indicates a potential structural relatedness of this hypothetical phage protein to several bacterial and archaeal proteins with DNA binding or transcription regulation functions (the best match is *Sulfolobus* DNA-binding protein Sul7s, PDB ID 7BZH_A). These results are compatible with the suggested involvement of the ORF14 protein product in CSg control. Interestingly, two out of three mutants with ORF14 alterations also had mutations in ORF3. This can indicate that the ORF14 modification alone is not enough for the clear CTg phenotype or that mutants affected in ORF14 are not permissible without compensatory mutations in ORF3.

The mutants CT2, CT5 and CT13 also carried large insertions (originating from duplication and replacement of neighboring sequences) in ORF25, which codes for DNA ligase. The involvement of ORF25 in the determination of the CSg phenotype also cannot be excluded, although it appears less likely since most of the cold-tolerant mutants described above grow at low temperature and are thus capable of correct synthesis of phage DNA.

## Conclusions

To summarize our data, we conclude that the restriction of bacteriophage AMP1 growth at low temperatures (ca. 25 °C) is not due to critical depletion of any viral or host function but results from the repression of productive phage infections by a phage-encoded mechanism. Our results allow us to conclude that ORF3 is involved in CSg determination. The data also strongly suggest that ORF14 protein also makes a part of this mechanism. Clearly, additional experimentation is required to unravel the exact details of how ORF3 and ORF14 proteins operate. The repression of AMP1 or related phage growth at low temperatures does not seem to be linked to lysogeny, at least under our experimental conditions, but instead causes slow or inefficient lytic growth of the phage.

## Supplementary Information

Supplementary Table S1.
