# Supplementary material for: Genetic analysis of the cold-sensitive growth phenotype of Burkholderia pseudomallei/thailandensis bacteriophage AMP1
Source: Sci Rep. 2022 Mar 11;12:4288. doi: 10.1038/s41598-022-07763-7 (PMC8917201; doi:10.1038/s41598-022-07763-7)
Supplement: Supplementary file 1 — Supplementary Table S1. [file 41598_2022_7763_MOESM1_ESM.docx]

| Mutants  ID | Polymorphisms | | | | | | | | | | |
| --- | --- | --- | --- | --- | --- | --- | --- | --- | --- | --- | --- |
|  | T55C | T567C | A1467C | 1615-16  ins C  cc => ccc | A1667C | del1688-1722  35bp | del1728-1733  6 bp | A1740G | ins4963-64  135bp | indels between  6531-78  resolve  sep | C6686T |
| CT2 | + | + | - | orf3  frameshift  after 47 aa | - | - | - | - | - | - | - |
| CT5 | + | + | - | - | - | - | - | orf3  D89G | - | orf14  RPQ72-75VRA,  del 76-91 | - |
| CT6 | + | + | SD orf3  agg**c**gg | - | - | - | - | - | orf10  ins 45 aa 67-68 | orf14  –//– | - |
| CT9 | + | + | - | - | - | orf3  del71-83, frameshift | - | orf3  D89G | - | orf14  73-91  repl by 48 aa | - |
| CT12 | + | + | - | - | orf3  T64P | - | - | - | - | - | + |
| CT13 | + | + | - | - | - | - | orf3  del84-85 (EC) | - | - | - | - |

|  | 16008-09 insertions | A20052G | ins25610-11  135 bp | G25777T | T26596G | A28482G | T32232C | C35787T | A37031G | T38042C |
| --- | --- | --- | --- | --- | --- | --- | --- | --- | --- | --- |
| CT2 | orf25  ins 129 bp  ins 43 a.a. | orf30  Q111R |  | TTP-B  silent | TTP-B  D481G | gp37 in vir pr  N208D |  | orf39  A147V | orf39  T549A |  |
| CT5 | orf 25 ligase  duplication  15898-16077  ins 45 aa | –//– |  | TTP-B |  | –//– |  | –//– | –//– | orf42  TerL  silent |
| CT6 | - | –//– |  | TTP-B |  | –//– | orf38  int vir pr  I658T | –//– | –//– |  |
| CT9 | - | –//– |  | TTP-B |  | –//– |  | –//– | –//– |  |
| CT12 | - | –//– | g35 – TTP-B  ins 32 aa  407-408 | TTP-B |  | –//– |  | –//– | –//– |  |
| CT13 | orf25  ins 135 bp  (two adjacent duplications) | –//– |  | TTP-B |  | –//– |  | –//– | –//– | –//– |

**Table S1.** The polymorphisms observed in the sequenced CT mutants genomes compared to the reference AMP1 phage sequence. Nucleotide coordinates are given according the reference sequence.
